# Clinical Outcomes and Predictors of Patients with Fracture in Debre Markos Comprehensive Specialized Hospital, North West Ethiopia: A Prospective Cohort Study

**DOI:** 10.1155/2022/3747698

**Published:** 2022-04-22

**Authors:** Beminet Yimenu, Belayneh Mengist

**Affiliations:** ^1^Department of Surgery, School of Medicine, Debre Markos University, Debre Markos, Ethiopia; ^2^Department of Public Health, College of Health Sciences, Debre Markos University, Debre Markos, Ethiopia

## Abstract

**Background:**

Fracture continues to be a major public health concern in many parts of the developing world that results in several consequences and complications including lifelong morbidity and mortality. This study aimed to assess clinical outcomes of patients following fracture in Debre Markos Comprehensive Specialized Hospital, North West Ethiopia.

**Methods:**

An institution-based prospective cohort study was conducted from November 2020 to July 2021 among 207 fracture patients (69 visited traditional bone setter and 138 did not visit traditional bone setter). Data were collected through face-to-face interviews, physical examinations, and radiological investigations. Data were entered using Epi-Data version 3.1 and analysis was done using STATA 14 statistical software. Descriptive statistics were summarized using mean, median, standard deviation, and percentage and presented in tables and figures. The generalized linear model was fitted to identify the risks of the outcome variable. Risk Ratio with its 95% confidence interval was used and factors with a *P*-value less than 0.05 were considered as a statistically significant association.

**Result:**

The mean age of the participants was 37.5 ± 13.6 years and two-thirds of the participants were males. Nearly half of the patients 92 (44%), 50 (54%) from the exposed and 42 (46%) from the nonexposed group, were delayed getting treatment from the hospital. The majority of the patients had been treated with Plaster of Paris immobilization (55%) followed by fixation (15%) and a combination of both (12%). Nearly half of the participants (48%), 74% from the exposed and 35% from the nonexposed group, developed complications during the follow-up period. The commonest complication was joint stiffness (45%) followed by osteoarthritis (21%). The risk of fracture-related complications among patients who did not visit traditional bone setter was decreased by 54% as compared to visitors (RR 0.46; 95% CI: (0.35, 0.60))

**Conclusion:**

The magnitude of complications following the fracture is found to be high and the risk of complications among patients who visited traditional bone setters increased significantly. Therefore, prevention measures should be strengthened and integration between hospitals and traditional bone setters should be made so that basic training on fractures management will be given.

## 1. Background 

A large number of traumatic injuries are orthopaedic in nature that cause damage to the musculoskeletal system, which includes bones, ligaments, joints, tendons, muscles, and nerves which can be traumatic as well as nontraumatic injuries [1]. Studies conducted on the outcome of trauma revealed that fracture is the commonest outcome of injuries [2].

Bone is relatively brittle, yet it has sufficient strength and resilience to withstand considerable stress. Most fractures are caused by sudden and excessive force, which may be direct or indirect [3].

Globally, injuries account for over 10% of disability-adjusted life-years, 90% of which occur in low-income and middle-income countries [4]. The epidemiology and spectrum fracture varies in different parts of the world and presentations to hospitals fluctuate considerably [2]. Injuries are common and increasing in most developing countries, including Sub-Saharan Africa [3]. According to World Health Organization (WHO) report, the rate of mortality associated with injury was highest in African nations of which Ethiopia was ranked third following Nigeria and South Africa [5]. Of musculoskeletal injuries, fractures represent 25% and more than 16.2 million fracture cases are treated each year [6].

Studies have shown that road traffic accidents (RTA) are the most common determinant of traumatic orthopaedic injuries, with a fracture prevalence of 29.4%, 49.3%, and 68.4% [1].

Fracture and dislocation have several consequences and complications including lifelong morbidity and mortality. Upper extremity ballistic injuries are common with intra-articular fractures resulting in more severe impact including lifelong morbidity, particularly in low resource areas where adequate trauma management is not available [7]. The main aim of fracture management is to obtain proper anatomic reduction, healing of the fracture, and restoration of physiology up to a maximum like preinjury state [8]. Traditional bone setting (TBS) is an old practice found in almost all areas of the world. Traditional bonesetters use different methods while treating these patients. Some use massage, traction, and splinting with strips of wood tightly bound to the limb occasionally including joints and bamboo sticks and they apply old clothes [8, 9]. Health workers, mainly the orthopaedic professionals in developing continents like Africa, face challenges caused by complications of fracture management by traditional bone setting practices [10]. The bone setting is one of the popular traditional medicines in Ethiopia, which is recognized to have attained a level of success comparable to that in modern medicine and the preference for the traditional bone setting is high although complications in the form of gangrene, nonunion, joint stiffness, and infections of limbs caused as a result of the traditional bone setting are quite common [11, 12]. Splints may not be removed when pain increases after immobilization leading to compartment syndrome with its permanent sequelae such as gangrene and death as a result of tetanus and septicemia [9].

In many parts of the developing world, fractures continue to be a major public health concern. It is treated by traditional bonesetters, who are readily available and often have a good local reputation. There are few orthopaedic medical facilities and patients have to travel as far to receive specialist surgical attention. Assessing the outcomes of patients following fracture using a strong design by taking traditional bone setter treatment as a comparable group is crucial. Therefore, this study aimed to assess clinical outcomes and predictors of patients with fracture who visited and did not visit traditional bone setters in Debre Markos Comprehensive Specialized Hospital, North West Ethiopia.

## 2. Methods

### 2.1. Study Design

A hospital-based prospective cohort study was employed. Patients with fracture who visited TBS and who did not visit TBS were taken as exposed and nonexposed groups, respectively.

### 2.2. Study Area and Period

This study was conducted at Debre Markos Comprehensive Specialized Hospital (DMCSH), the only referral hospital in the East Gojjam zone and found in Debre Markos; the town of the administrative zone is found 299 kilometers North West of Addis Ababa. The hospital serves more than 3.5 million populations and it is the only hospital providing orthopaedic service in the East Gojjam zone. The orthopaedic unit shares 36 beds with general surgery and three operation tables with general surgery, gynaecology, and obstetrics. Patients with a displaced fracture are supposed to be admitted and fixation is done, either internal or external fixation. Those patients with nondisplaced fractures and noncomplicated cases are treated on an outpatient basis.

This study was conducted from November 2020 to July 2021.

### 2.3. Population

#### 2.3.1. Source Population

All adult patients who had fracture during the study period in the hospital were considered as the source population.

#### 2.3.2. Study Population

Adult patients admitted to the surgical ward with fracture and those who visited the emergency department in the hospital during the study period were the study population.

### 2.4. Eligibility Criteria

#### 2.4.1. Inclusion Criteria

Adult patients admitted to the surgical ward with fracture during the study period and those who had visited the emergency department of the hospital were included.

#### 2.4.2. Exclusion Criteria

Adult patients with pathologic fracture and patients with fracture having other concomitant body injuries were excluded. In addition, patients who presented with the complication at the beginning of the study were excluded.

### 2.5. Sample Size Determination and Procedure

#### 2.5.1. Sample Size Determination

To determine sample size, various predictors significantly associated with the outcome variable were considered. Accordingly, the sample size was determined using the double population proportion formula by taking the nature of fracture as a variable from a previous study [2] using Epi Info version 7 statistical software by considering the following parameters:

- P1: proportion of exposed with the outcome (0.23)
- P2: proportion of nonexposed with the outcome (0.07)
- *Z α*/2 :  95% confidence level
- *Zβ*: power = 80%
- *r* is the ratio of exposed to nonexposed group = 1

The sample size was determined to be 180; by adding 15% withdrawal probability, the final sample size was 207.

#### 2.5.2. Sampling Procedure

The study participants were selected systematically from the surgical ward and emergency department of the hospital during the study period. Then, exposure status was determined using face-to-face interviews whether they visited TBS or not. Finally, the selected patients were followed till the declaration of clinical outcomes. Each patient had at least three follow-up times and frequent phone reminders were made.

### 2.6. Variable of the Study

#### 2.6.1. Dependent Variable

- clinical outcomes of fracture.

#### 2.6.2. Independent Variables

- socio-demographic characteristics (age, sex, educational status, occupation, and place of residence).
- Clinical predictors (types of fracture, comorbidity, delays, and visiting TBS) were considered.

### 2.7. Operational Definitions

- Delay in hospital care is defined as more than 2 hours from the time of injury to visit the hospital for an open fracture and more than 24 hours for those with closed fractures [4].
- The outcome of fracture was defined as good if the patient was discharged without complication and poor if the patient developed at least one complication.

### 2.8. Data Collection Tool and Procedure

A structured data collection tool was developed in English by considering study variables from different works of literature. The checklist consists of socio-demographic characteristics (age, marital status, employment status, educational level, and residence) and clinical characteristics (types of fracture, comorbidity, delays, types of complication, and visit TBS). Data were collected from selected patients during the follow-up period. Outcomes of fracture patients were assessed by interviewing, doing physical examination on patients, and X-ray imaging. Three trained physicians working at the ward were recruited for data collection and two investigators supervised the overall data collection process.

### 2.9. Data Quality Assurance

Data were collected from study participants by trained data collectors and close supervision of the entire data collection process was done. The two-day training was given for data collectors concerning the data collection tool and data collection process. Data quality was also assured by designing a proper data collection tool. All collected data were checked for completeness and clarity by the data collector and supervisor every day.

### 2.10. Data Processing and Analysis

Data were entered using Epi-Data version 3.1 and analysis was done using STATA 14 statistical software. Data were cleaned and edited before analysis. Descriptive statistics were summarized using mean, median, standard deviation, and percentage presented in tables and figures as appropriate. The generalized linear model was fitted by taking risk ratio (RR) as a measure of association to identify potential risk factors of the outcome. Both deviance and Bayesian information criteria (BIC) were used to select the best-fitted model. RR with its 95% confidence interval was used, and with a *p* value less than 0.05 were considered as a statistically significant association.

### 2.11. Ethical Consideration

The ethical issue was taken into consideration when carrying out the study. Ethical approval was obtained from the ethical review committee of Debre Markos University (DMU), School of Medicine. A formal letter was submitted to DMCSH and permission was assured and informed consent was obtained from each patient. To keep confidentiality, names and medical registration numbers were not included in the data collection format and the data were not disclosed to any person other than investigators. All information collected from the patients was kept strictly confidential and was not used for another purpose.

## 3. Result

### 3.1. Socio-Demographic Characteristics of Participants

A total of 207; 69 (33%) exposed and 138 (67%) nonexposed patients with fractures, were included in the study. The mean age of the participants was 37.5 ± 13.6 years and the proportion of male participants was two-thirds of the total participants (65%); 58% were from the exposed and 77% were from the nonexposed group. The majority of the participants were orthodox religion followers (87% from exposed and 94% from nonexposed) and half of the participants were farmers (39% from exposed and 54% from nonexposed) (Table 1).

### 3.2. Clinical Characteristics of Patients

All study participants were followed until the outcome was declared by an orthopaedic surgeon supported with X-ray images. The majority of the patients had been treated with Plaster of Paris (POP) immobilization (55%) followed by fixation (15%) and a combination of both (12%). Nearly half of the participants (48%), 74% from the exposed and 35% from the nonexposed group, developed complications during the follow-up period (Table 2).

From visitor patients, 93% were visited TBS because of traditional beliefs and the rest were due to cost and fear of amputation. The mean duration of stay at TBS was 8 ± 10 days. Nearly half of the patients 92 (44%), 50 (54%) from the exposed and 42 (46%) from the nonexposed group, were delayed getting treatment from the hospital. The major reason for the delay was because of the TBS visit (48%) followed by lack of transportation which accounts for 28%. The commonest complication was joint stiffness (45%) followed by osteoarthritis (21%). One-third of the participants (35%) had two or more complications throughout the follow-up time (Table 3).

### 3.3. Factors Associated with Clinical Outcomes of Patients with Fracture

Variables that have great clinical and statistical importance (age, sex, residence, visiting TBS, delay to receive care, presence of comorbidity, and type of fracture) were assessed using a generalized linear model. Visiting TBS was significantly associated with patients' outcomes; that is, patients with a fracture who visited TBS had a higher risk to develop complications. The risk of fracture-related complications among patients who did not visit TBS was decreased by 54% as compared to visitors (RR 0.46; 95% CI: (0.35, 0.60)) (Table 4).

## 4. Discussion

Studies reported that there is a consistent increase in the number of traumatic emergencies which result in fractures. The aim of this study was to assess the clinical outcomes of patients who visited the hospital following fracture which is new in its type, particularly in the study area. The study revealed that the mean age of the participants was 37.5 ± 13.6 years and the proportion of male participants was two-thirds of the total participants and half of the participants were farmers. This demographic trend is similar to the previous studies done in Ethiopia, Wolaita Sodo [2] and Addis Ababa [13]. This indicates that a higher proportion of the economically productive age group is affected. The higher burden among males and farmers is probably due to the nature of work they are participating in.

One-third of the participants visited TBS and the majority of them (93%) visited because of traditional beliefs followed by fear of cost and amputation. This finding is consistent with the previous finding from Black Lion Hospital in Addis Ababa, Ethiopia [12]. This is likely due to the presence of strong cultural beliefs of the population in the study area. A study in Kenya also indicated that the cheaper fees requested by TBS increase the belief and reliance on treatment by TBS practitioners [14]. In addition, the possibility of a payment in the form of a kind such as cloth and animals could be another driving force [15–18]. Bamboo splinting, massaging, splinting by homemade materials, and application of herbal medicines were the commonly used practices for the treatment of fracture by TBS. These practices are also implemented by other African countries such as Sudan [6], Nigeria [15], Tanzania [17], and Ghana [19].

Twenty-eight percent of the patients were delayed to get treatment from the hospital due to difficulty to reach care, that is, lack of transportation. This finding is supported by a study from low-income and middle-income countries [4]. This is a result of the low accessibility of infrastructures, particularly in rural settings.

The majority of fractures reported were compound type (56%) and dominantly happened in the lower limbs which is consistent with previous study findings [2, 16] that is because of its anatomic susceptibility.

The study showed that most of the fractures were occurred due to RTA (30.4%) which is consistent with previous studies conducted in Ethiopia; Jimma (30.3) [20], Wolaita Sodo (40.2%) [21], (35%) [2], and Nigeria (45.8%) [16] need further investigation.

The magnitude of complications associated with fracture was determined; nearly half of the patients (47.8%) developed a complication. This finding is higher than the previous study conducted in Wolaita Sodo, Ethiopia (22.5%) [2]. This discrepancy might be due to the difference in the study period (2015/2016) and the study design used. The proportion of complications among the exposed (visited TBS) group is higher than the nonexposed group (not visited TBS). This finding is consistent with studies conducted in Ethiopia (56.9%). The commonest complication resulting from fracture was joint stiffness (45%) which is similar to the previous study [21] followed by osteoarthritis (21%).

The analytical component of this study showed that visiting TBS was significantly associated with patients' outcomes, that is, patients with a fracture who visited TBS had a higher risk to develop a complication. The risk of fracture-related complications among patients who did not visit TBS was decreased significantly. This finding is supported by previous studies [2, 8, 21]. This is because TBSs have no basic knowledge of anatomy, physiology, imaging, and principles of infection prevention and control practices [8].

## 5. Conclusion and Recommendation

A road traffic accident is the commonest cause of fracture and a significant number of patients have arrived in the hospital lately. The magnitude of complications following fracture is found to be high. Moreover, the risk of complications among patients who visited TBS was significantly high.

Therefore, prevention measures should be strengthened to reduce road traffic accidents and early referral of fracture patients to facilities that have orthopaedist shall be facilitated. Integration between hospitals and TBS should be made so that basic training on fractures management will be given. Since the hospitals providing orthopaedic services are few and far away from most of the people living in rural settings, the TBS practitioners should be linked with modern health system.

## Figures and Tables

**Table 1:** Socio-demographic characteristics of patients with fracture in DMCSH, 2021.

Variables	Category	Exposed frequency (%)	Nonexposed frequency (%)	Total frequency (%)
Age	<30 years old	22 (31.9)	54 (39.1)	76 (36.7)
	≥30 years old	47 (68.1)	84 (60.9)	131 (63.3)

Sex	Male	40 (57.9)	95 (77.2)	135 (65.2)
	Female	29 (42.1)	43 (22.8)	72 (34.8)

Religion	Orthodox	60 (87.0)	130 (94.2)	190 (91.8)
	Muslim	7 (10.1)	3 (2.2)	10 (4.8)
	Protestant	2 (2.9)	5 (3.6)	7 (3.4)

Residence	Rural	36 (52.2)	92 (66.7)	128 (61.8)
	Urban	33 (47.8)	46 (33.3)	79 (39.2)

Education level	No formal education	28 (40.6)	66 (47.8)	94 (45.4)
	Primary school	15 (21.7)	32 (23.2)	47 (22.7)
	Secondary school	14 (20.3)	15 (10.9)	29 (14.0)
	Higher education	12 (17.4)	25 (18.1)	37 (17.9)

Marital status	Single	17 (24.6)	26 (18.8)	43 (20.8)
	Married	46 (66.7)	99 (71.7)	145 (70.1)
	Divorced	3 (4.3)	6 (4.3)	9 (4.3)
	Widowed	3 (4.3)	7 (5.1)	10 (4.8)

Occupation	Gov't employed	6 (8.7)	16 (11.6)	22 (10.6)
	Housewife	8 (11.6)	2 (1.4)	10 (4.8)
	Farmer	27 (39.1)	75 (54.3)	102 (49.3)
	Merchant	14 (20.3)	27 (19.6)	41 (19.8)
	Student	8 (11.6)	9 (6.5)	17 (8.2)
	Others	6 (8.7)	9 (6.5)	15 (7.3)

**Table 2:** Clinical characteristics of patients with fracture in DMCSH, 2021.

Variables	Category	Exposed frequency	Nonexposed frequency	Total frequency (%)
Mode of arrival	Ambulance	9	56	65 (31.4)
	Public transport	57	80	137 (66.1)
	Traditional carry	3	2	5 (2.5)

Treatment category	Inpatient	24	95	119 (57.5)
	Outpatient	45	43	88 (42.5)

History of previous fracture	Yes	16	16	32 (15.5)
	No	53	122	175 (85.5)

Delay to receive care	Yes	50	42	92 (44.4)
	No	19	96	115 (55.6)

Types of fracture	Closed	39	52	91 (44.0)
	Compound	30	86	116 (56.0)

Fracture site	Upper limb	32	49	81 (39.1)
	Lower limb	32	60	92 (44.4)
	Upper and lower	2	27	29 (14.0)
	Others	3	2	5 (2.5)

Number of fractures	One	54	82	136 (65.7)
	Two	12	36	48 (23.2)
	Three and above	3	20	23 (11.1)

Mechanism of injury	RTA	16	47	63 (30.4)
	Failing accident	33	18	51 (24.6)
	Stick injury	11	41	52 (25,1)
	Bullet injury	4	25	29 (14.0)
	Others	5	7	12 (5.7)

Condition during arrival	Critical	18	92	110 (53.1)
	Stable	51	46	97 (46.9)

Comorbidity	Yes	13	19	32 (15.5)
	No	56	119	175 (84.5)

Complication	Yes	51	48	99 (47.8)
	No	18	90	108 (52.2)

**Table 3:** TBS-related information and complications among patients with fracture in DMCSH, 2021.

Variables	Category	Frequency (%)
Reason to visit TBS	Traditional belief	64 (92.7)
	Low cost	3 (4.4)
	Fear of amputation	2 (2.9)

Treatment given by TBS	Bamboo splinting	27 (39.1)
	Massaging	16 (23.2)
	Massaging and splinting by cloth	17 (24.6)
	Herbal medicine and others	9 (13.1)

Types of complication^*∗*^	Joint stiffness	45 (45.5)
	Soft tissue infection	21 (21.2)
	Osteoarthritis	15 (15.2)
	Nonunion	9 (9.1)
	Malunion	7 (7.1)
	Osteomyelitis	7 (7.1)
	Compartment syndrome	5 (5.0)
	Volkmann's contracture	2 (2.3)

^*∗*^Some patients had multiple complications.

**Table 4:** Multivariable regression analysis of outcome of patients with fracture in DMCSH, 2021.

Variables	Category	CRR (95% CI)	ARR (95% CI)	*P* value
Age	<30 years	Ref	Ref	0.87
	≥30 years	1.11 (0.82, 1.50)	1.09 (0.80, 1.48)	

Sex	Male	Ref	Ref	0.29
	Female	0.98 (0.72, 1.32)	0.86 (0.66, 1.13)	

Residence	Rural	Ref	Ref	0.085
	Urban	0.92 (0.68, 1.24)	0.78 (0.60, 1.03)	

TBS	Visitor	Ref	Ref	<0.001^*∗*^
	Nonvisitor	0.47 (0.36, 0.61)	0.46 (0.35, 0.60)	

Delay to receive care	Yes	Ref		0.09
	No	0.74 (0.59, 1.04)	0.78 (59.1.04)	

Type of fracture	Closed	Ref	Ref	0.62
	Compound	1.02 (0.76, 1.36)	1.06 (0.83, 1.35)	

Comorbidity	Yes	Ref	Ref	0.18
	No	0.76 (0.55, 1.07)	0.83 (0.64, 1.09)	

## Data Availability

The datasets used and/or analyzed during the current study are available from the corresponding author on reasonable request.

## Abbreviations

DMCSH: Debre Markos Comprehensive Specialized Hospital
RTA: Road traffic accidents
TBS: Traditional bone setting
WHO: World Health Organization.

## References

[1] Ghani D. A. (2018). An analytical study of pattern of orthopedic injuries among patients presenting to the emergency department in a tertiary care hospital at GMC jammu. *Journal of Medical Science and clinical Research*.

[2] Bayisa J. G. E. T. H. (2017). Factors associated with fracture and its outcome at Wolaita Sodo university teaching and referral hospital, Wolaita Sodo, southern Ethiopia. *Journal of Biology, Agriculture and Healthcare*.

[3] Nayagam L. D. W. S. (2010). *Apley’s System of Orthopedics and Fracture*.

[4] Pouramin P., Li C. S., Busse J. W. (2020). Delays in hospital admissions in patients with fractures across 18 low-income and middle-income countries (INORMUS): a prospective observational study. *Lancet Global Health*.

[5] Word Health Organization (WHO) (2004). *Measurement and Health Information Data Sheet*.

[6] Idris S. M. O. B., Basheer E. S. (2010). Why do people prefer traditional bonesetters in Sudan?. *Sudan Journal of Medical Sciences*.

[7] Esmee Wilhelmina S. R. M. E., Maqungo S., Naude D., Held M. (2019). Treating fractures in upper limb gunshot injuries: the Cape Town experience. *Orthopaedics & Traumatology: Surgery & Research*.

[8] Alam W., Shah F. A., Ahmed A., Ahmad S., Shah A. (2016). Traditional bonesetters. *The Professional Medical Journal*.

[9] Eshete M. (2005). The prevention of traditional bone setter’s gangrene. *The Journal of Bone and Joint Surgery*.

[10] Chidera O. F. (2018). *Traditional Bone-Setting Procedures Intrue Fracture in Imo State*.

[11] Woyessa A. H., Dibaba B. Y., Hirko G. F., Palanichamy T. (2019). Spectrum, pattern, and clinical outcomes of adult emergency department admissions in selected hospitals of western Ethiopia: a hospital-based prospective study. *Emergency Medicine International*.

[12] Nardos Worku T. T., Abdissa B., Merga H. (2019). Preference of Traditional Bone Setting and associated factors among trauma patients with fracture at Black Lion Hospital in Addis Ababa, Ethiopia: institution based cross sectional study. *BMC Research Notes*.

[13] Seid M., Azazh A., Enquselassie F., Yisma E. (2015). Injury characteristics and outcome of road traffic accident among victims at Adult Emergency Department of Tikur Anbessa specialized hospital, Addis Ababa, Ethiopia: a prospective hospital based study. *BMC Emergency Medicine*.

[14] Maj S. J., Emma M. K. (2020). A study on fracture management by traditional bonesetters and its effects to clients in Lungalunga constituency, Kwale county in Kenya.

[15] Dada A. A. (2011). Review of the practice of traditional bone setting in Nigeria. *African Health Sciences*.

[16] Odatuwa-Omagbemi D. O., Adiki T. O., Elachi C. I., Bafor A. (2018). Complications of traditional bone setters (TBS) treatment of musculoskeletal injuries: experience in a private setting in Warri, South-South Nigeria. *The Pan African Medical Journal*.

[17] Card E. B., Obayemi J. E., Shirima O. (2020). Practices and perspectives of traditional bone setters in northern Tanzania. *Annals of Global Health*.

[18] Yempabe T., Edusei A., Donkor P., Buunaaim A., Mock C. (2021). Factors affecting utilization of traditional bonesetters in the Northern Region of Ghana. *African Journal of Emergency Medicine*.

[19] Yempabe T., Edusei A., Donkor P., Buunaaim A., Mock C. (2020). Traditional bonesetters in northern Ghana: opportunities for engagement with the formal health sector. *Pan African Medical Journal*.

[20] Woldemichael K., Berhanu N. (2011). Magnitude and pattern of injury in Jimma university specialized hospital, South West Ethiopia. *Ethiopian Journal of Health Sciences*.

[21] Wondimagegn P., Kumma B. Y. K. E. W. W. (2013). Complications of fracture treatment by traditional bone setters in Wolaita Sodo, southern Ethiopia. *Journal of Biology, Agriculture and Healthcare*.

